# Beta-band differences in primary motor cortex between media and non-media professionals when watching motor actions in movies

**DOI:** 10.3389/fnins.2023.1204809

**Published:** 2023-06-26

**Authors:** Celia Andreu-Sánchez, Miguel Ángel Martín-Pascual, Agnès Gruart, José María Delgado-García

**Affiliations:** ^1^Neuro-Com Research Group, Department of Audiovisual Communication and Advertising, Universitat Autònoma de Barcelona, Barcelona, Spain; ^2^Research and Innovation, Institute of Spanish Public Television (RTVE), Corporación Radio Televisión Española, Barcelona, Spain; ^3^Division of Neurosciences, University Pablo de Olavide, Sevilla, Spain

**Keywords:** movies, motor action, beta band, brain activity, neurocinematics

## Abstract

To watch a person doing an activity has an impact on the viewer. In fact, the film industry hinges on viewers looking at characters doing all sorts of narrative activities. From previous works, we know that media and non-media professionals perceive differently audiovisuals with cuts. Media professionals present a lower eye-blink rate, a lower activity in frontal and central cortical areas, and a more organized functional brain connectivity when watching audiovisual cuts. Here, we aimed to determine how audiovisuals with no formal interruptions such as cuts were perceived by media and non-media professionals. Moreover, we wondered how motor actions of characters in films would have an impact on the brain activities of the two groups of observers. We presented a narrative with 24 motor actions in a one-shot movie in wide shot with no cuts to 40 participants. We recorded the electroencephalographic (EEG) activity of the participants and analyzed it for the periods corresponding to the 24 motor actions (24 actions × 40 participants = 960 potential trials). In accordance with collected results, we observed differences in the EEG activity of the left primary motor cortex. A spectral analysis of recorded EEG traces indicated the presence of significant differences in the beta band between the two groups after the onset of the motor activities, while no such differences were found in the alpha band. We concluded that media expertise is related with the beta band identified in the EEG activity of the left primary motor cortex and the observation of motor actions in videos.

## 1. Introduction

### 1.1. Looking at motor actions

We perceive various narrative contents in plenty of motor actions through the day, and the observation of those actions has an impact on our brain activity (Muthukumaraswamy et al., 2004). It was more than half a century ago that an “arch rhythm” (with spectral peaks at 10 and 20 Hz) was found when looking at motor actions in movies (Gastaut et al., 1952; Cohen-Séat et al., 1954; Gastaut and Bert, 1954). One of the motor actions most studied in recent neuroscience has been grasping (Castiello, 2005). In this regard, it has been reported that human electroencephalographic mu rhythm (~8–13 Hz) changes while observing other people doing motor activities such as grasping, holding, and tearing (di Pellegrino et al., 1992; Gallese et al., 1996; Muthukumaraswamy et al., 2004). Moreover, previous experience in performing specific tasks seems to be more influential on this mu rhythm than observation of the task itself (Cannon et al., 2014). Other brain rhythms have also been studied in relation to grasping and action movements, such as alpha (~8–12 Hz; Perry and Bentin, 2010) or beta (~13–35 Hz) rhythms (Zaepffel et al., 2013; Khanna and Carmena, 2015). Several studies have worked on revealing neural correlates of grasping and other hand movements when doing them (Chavarriaga et al., 2018), when imaging them (Neuper et al., 2005; Ying et al., 2017), or when looking at someone doing them (Perry and Bentin, 2009). There are also studies that have compared activity modulation of brain EEG recordings while producing or observing social actions (Liao et al., 2015). Most of the studies about visual perception of motor activities paid attention to the primary motor cortex, suggesting a research interest in how a perceiver’s brain activity is modulated in this area by the motor activity developed by the actor of the content. In the present study, we recorded the EEG activity in the primary motor cortex of media and non-media professionals watching a one-shot video showing different motor actions of an actor participating in the movie.

### 1.2. Professional expertise

Professionalization has been shown to be a relevant element when motor brain activity is the subject of study. For instance, professional athletes learn complex dynamic visual scenes better than non-athletes do (Faubert, 2013), and professional racing-car drivers show an increased neural efficiency in brain circuits as compared with naïve drivers (Bernardi et al., 2013). Furthermore, evidence has been found regarding music expertise, including that brain structures differ between musicians and non-musicians (Gaser and Schlaug, 2003), piano players seem to need more-reduced neuronal networks than control subjects to activate the same movements (Krings et al., 2000), musical training has been associated with an altered processing of negative emotions (Park et al., 2014), and professional musicians show more-focused cerebral activations in the contralateral primary sensorimotor cortex (Lotze et al., 2003). There are also studies regarding the impact, in terms of brain activity, of esthetics expertise (Kirk et al., 2008), baseball expertise (Muraskin et al., 2016), football professionalization (Brockhoff et al., 2011), or dancing professionalization (Calvo-Merino et al., 2005), among others. These studies are focused on the neuroscience of expertise, in a context where the performance of an activity becomes more efficient and automatic, proving a perceptual expertise that relies on information from the senses (Bilalić, 2017), and showing how it is of interest to keep researching on cognitive and motor expertise in order to learn how we can improve motor actions.

We have previously studied the impact of media professionalization in visual perception of movies. First, we studied the spontaneous blink rate (SBR) in media professionals and non-media professionals while they were watching movies and looking at theatrical narrative performances, and we found a significant inhibition of SBR in the professional group (Andreu-Sánchez et al., 2017). We found that—since SBR is inversely linked to attention (lower SBR correlates with higher attentional level)—media expertise evokes a higher attention to narratives in both on-screen and live performances. In addition, we checked that media professionalization impacts cognitive neurodynamics during audiovisual cuts: while cuts in movies trigger similar activation of visual cortex, differences are found in central and frontal cortical areas, with a lower activity among media professionals (Andreu-Sánchez et al., 2021). Moreover, after the new visual information that cuts bring to spectators, effective brain connectivity is more organized in media professionals than in non-media professionals (Andreu-Sánchez et al., 2021).

In the present study, we wondered how media professionals’ brain activity in primary motor cortex would differ from that of non-media professionals when they were watching motor actions on screens, in a video without cuts that could interfere in the perceptive process.

## 2. Materials and methods

### 2.1. Participants

Forty participants aged 28–56 (43.75 ± 7.837) took part in this study. The group of media professionals (*N* = 20) comprised 15 males and five females. Their mean age was 44.25 ± 7.196 years. The time spent in their media professions was 20.2 ± 8.637 years. The group of non-media professionals (*N* = 20) consisted of 16 males and four females. Their mean age was 43.25 ± 8.589 years. The time spent in their non-media professions was 18.85 ± 9.422 years. Inclusion in the media professional group required participants to use video edition and to take decisions related to media editing in their everyday work. Non-media professionals were chosen outside of this criterion. All had normal or corrected-to-normal visual acuity. Subjects did not receive any economic compensation for their participation in this study.

### 2.2. Ethics statement

The studies involving human participants were reviewed and approved by the Ethics Commission for Research with Animals and Humans (CEEAH) of the Universitat Autònoma de Barcelona (Barcelona, Spain). The participants provided their written informed consent to participate in this study.

### 2.3. Stimuli and procedure

We created four stimuli with the same narrative but different formats: (1) a one-shot movie in wide shot with no camera movements; (2) a movie edited according to classical rules of edition with smooth transitions; (3) a movie edited breaking classical rules of edition, having a chaotic style with sharp and illogical transitions between shots; and (4) a live performance. All four stimuli were randomly presented to all participants, but, for the purpose of this study, we only analyze the one-shot movie with no cuts. Thus, we isolate the effect of viewing video content without breaks due to editing cuts. The selected movie had a duration of 198 s. The narrative included 24 motor actions that were used as triggers to analyze participants’ brain activities. The selected motor actions included objects being grasped, caught, and gripped.

Video stimuli were presented on a 42-in HD Led display (Panasonic TH-42PZ70EA) and participants were placed at 150 cm from the screen. Stimuli were presented with Paradigm Stimulus Presentation (Perception Research System Inc.). Participants were asked to attend to the stimuli, with no further information being given that a follow-up questionnaire would be presented. The questionary presented was actually a distractor without interest for the research.

### 2.4. Data acquisition

Continuous EEG data were acquired with the help of a wireless system (Enobio, Neuroelectrics), with 20 electrodes placed according to the International 10–20 system [O1, O2, P7, P3, Pz, P4, P8, T7, C3, Cz, C4, T8, F7, F3, Fz, F4, F8, Fp1, Fp2, and an external electrode used for electrooculogram (EOG) recording] referenced to electronically linked mastoid electrodes (see Martín-Pascual et al., 2018 for details). Data were sampled at 500 Hz. We recorded facial expressions of participants with an HD video-camera for contrasting participants’ behavior during the sessions, and additionally to detect and to avoid artifacts and unwanted muscle movements.

### 2.5. Data analysis

Electroencephalographic data were processed using EEGLAB (Delorme and Makeig, 2004) software version 2022.1 running on MATLAB R2022b (The MathWorks Inc.) under a macOS Ventura 13.2.1 (Apple Inc.). We band-passed filtered the data between 0.5 and 40 Hz. We removed the EOG electrode and bad channels when needed. A common average reference was applied. We decomposed data with an ICA analysis (infomax algorithm) and got rid of artifactual components, including eye and muscle activity (Delorme and Makeig, 2004). We made 3-s epochs of 1 s before and 2 s after the onset of the motor activity, marked with triggers at the onset of the motor actions in the video. In total, we had 40 participants who attended 24 motor actions, making 40 × 24, i.e., 960 potential trials of 3 s each. We rejected bad epochs through visual inspection.

We analyzed data recorded from the primary motor cortex of both hemispheres. The left hemisphere was studied with activity from the C3 electrode, and the right hemisphere was studied with activity from the C4 electrode. We computed spectral activity in alpha (8–12 Hz) and beta (13–30 Hz) bands. We also distinguished between activity before (−500–0 ms) and after (0–1,000 ms) the onset of the motor activity. Statistical analysis was performed offline using JASP software (Version 0.17.1, Apple Silicon). We computed unpaired *t*-test or non-parametric Mann–Whitney Rank Sum Tests for each situation. Effect size was computed with rank-biserial correlation. For the normality test, we used the Shapiro–Wilk. We also computed event-related spectral perturbation (ERSP) and power spectrum density (PSD) for those C3 and C4 electrodes and compared them among the groups. We also computed event-related spectral perturbation (ERSP) and power spectrum density (PSD) for those C3 and C4 electrodes and compared them among the groups. ERSP (Delorme and Makeig, 2004) is used to visualize mean event-related changes in spectral power over time in a broad frequency range and generalize the narrow-band event-related desynchronization (ERD) and synchronization (ERS) measures introduced by Pfurtscheller and Aranibar (1979). And PSD helps to know how the strength of a signal is distributed in the frequency domain and its unit is energy per frequency, demonstrating the strength of the variations of a signal as a function of frequency (Valipour et al., 2014). We also plot the inter-trial coherence (ITC). We computed t-tests with a significance of *p* < 0.05 with EEGLAB statistics running on MATLAB.

## 3. Results

**Figure 1:**
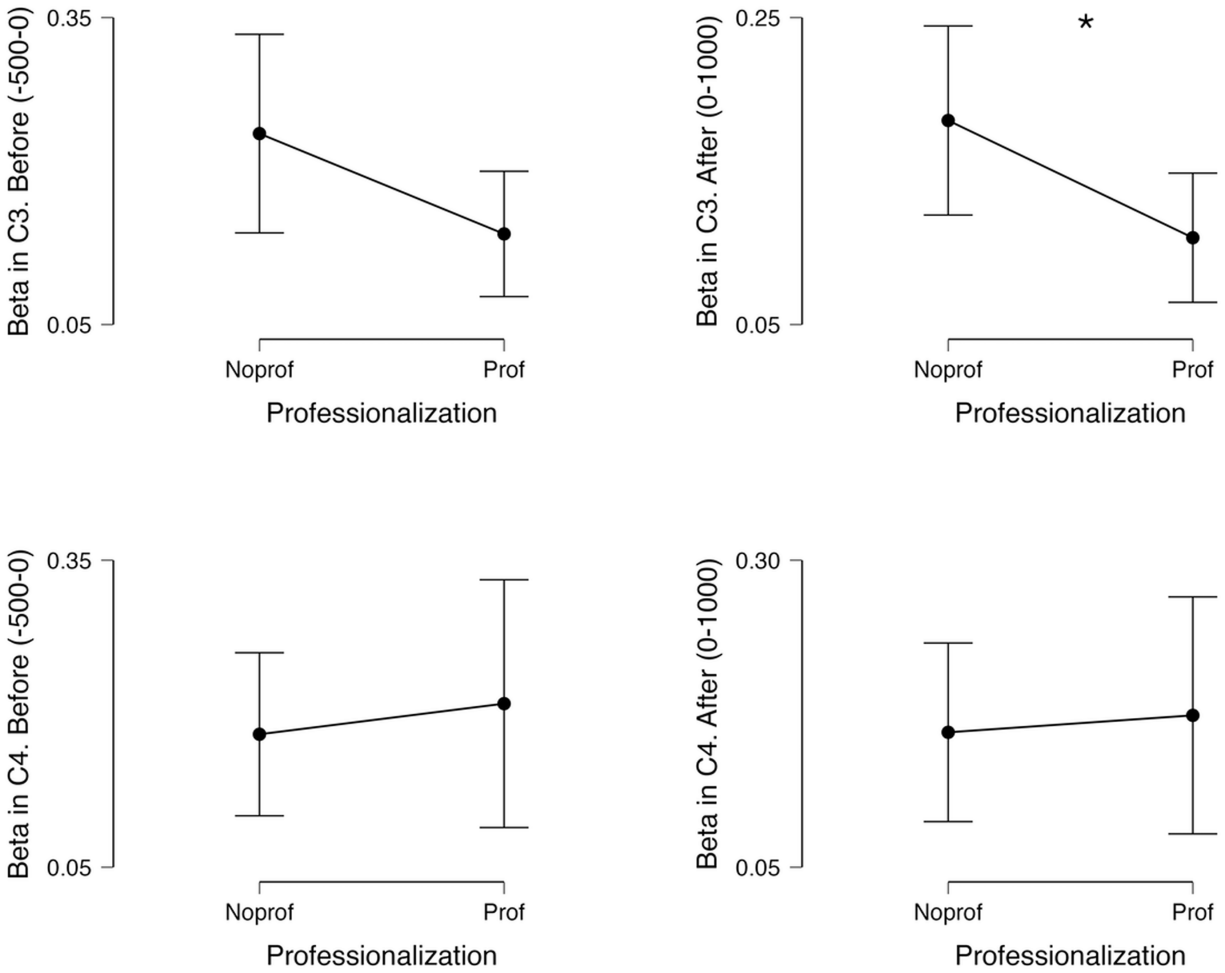
Mean spectral power in the beta band in media and non-media professionals, before the onset of the motor activities within the video (−500 to 0 ms) and after the onset of the motor activities (0–1,000 ms), in left (C3) and right (C4) hemispheres. ^*^indicates *p* < 0.05, non-parametric Mann–Whitney test.

In the case of beta band (13–30 Hz) comparison between media and non-media professionals, although we did not find significant differences before the onset of the motor actions in the left hemisphere (C3 electrode; U = 264, *p* = 0.086, Mann–Whitney test, rank-biserial correlation: 0.320), we did find significant differences after the onset of the motor activities in C3: U = 273, *p* = 0.049, Mann–Whitney test, rank-biserial correlation: 0.365. In contrast, the right hemisphere (C4 electrode), did not show significant differences before (U = 191, *p* = 0.820, Mann–Whitney test, rank-biserial correlation: −0.045) or after the onset of the motor activities (U = 191, *p* = 0.820, Mann–Whitney test, rank-biserial correlation: −0.045; see Figure 1). In addition, we did not find significant differences in the alpha band (8–12 Hz) between groups in either the left hemisphere (C3 electrode) before (U = 214, *p* = 0.718, Mann–Whitney test, rank-biserial correlation: 0.070) and after (U = 219, *p* = 0.620, Mann–Whitney test, rank-biserial correlation: 0.095) the onset of the motor actions, or the right hemisphere (C4 electrode) before (U = 164, *p* = 0.341, Mann–Whitney test, rank-biserial correlation: −0.180) and after (U = 171, *p* = 0.341, Mann–Whitney test, rank-biserial correlation: −0.145) the onset of the motor actions.

**Figure 2:**
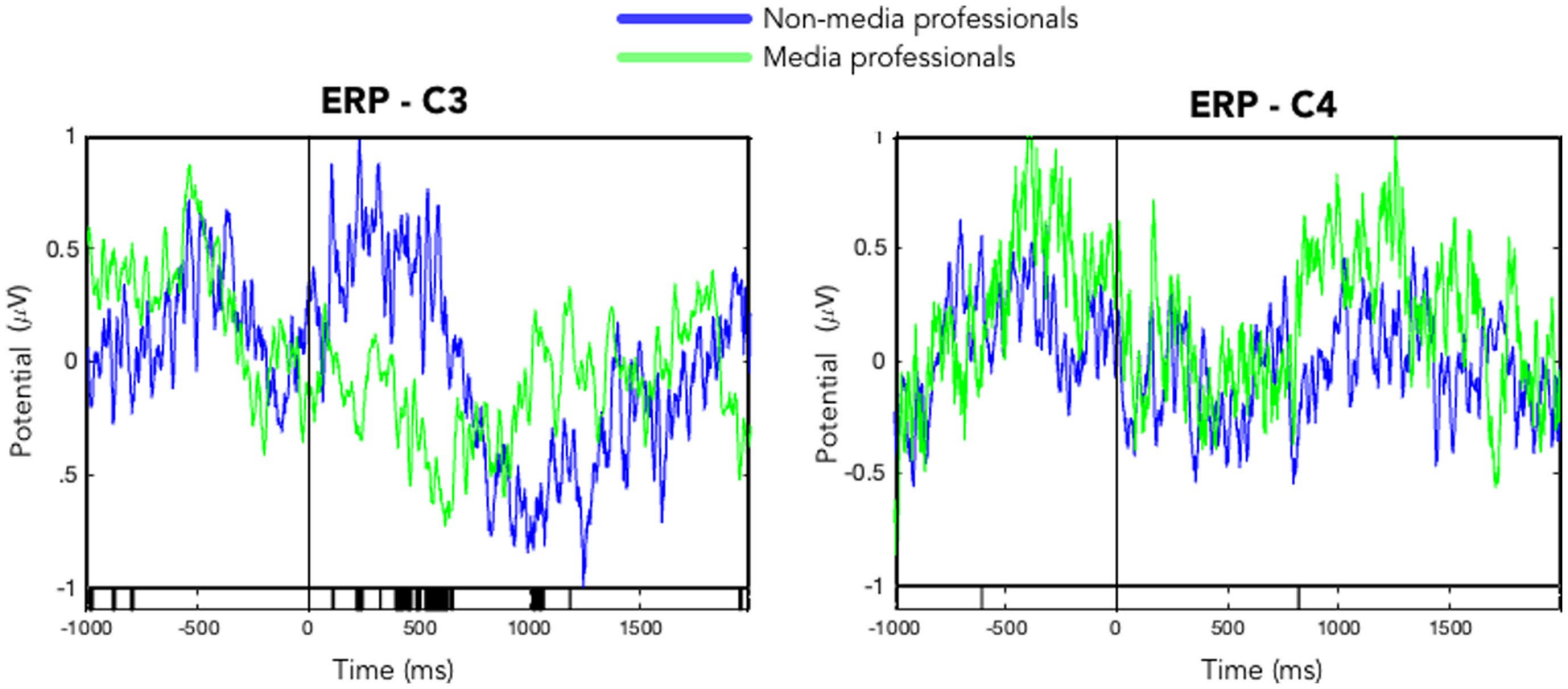
Average ERSPs of C3 and C4 in media (green) and non-media (blue) professionals, while watching 24 motor actions in the video. Vertical black lines (at Time 0) indicate the onset of the motor action of the character in the video. The lower vertical black bars show significant differences between groups across time (*t*-test, *p* < 0.05).

**Figure 3:**
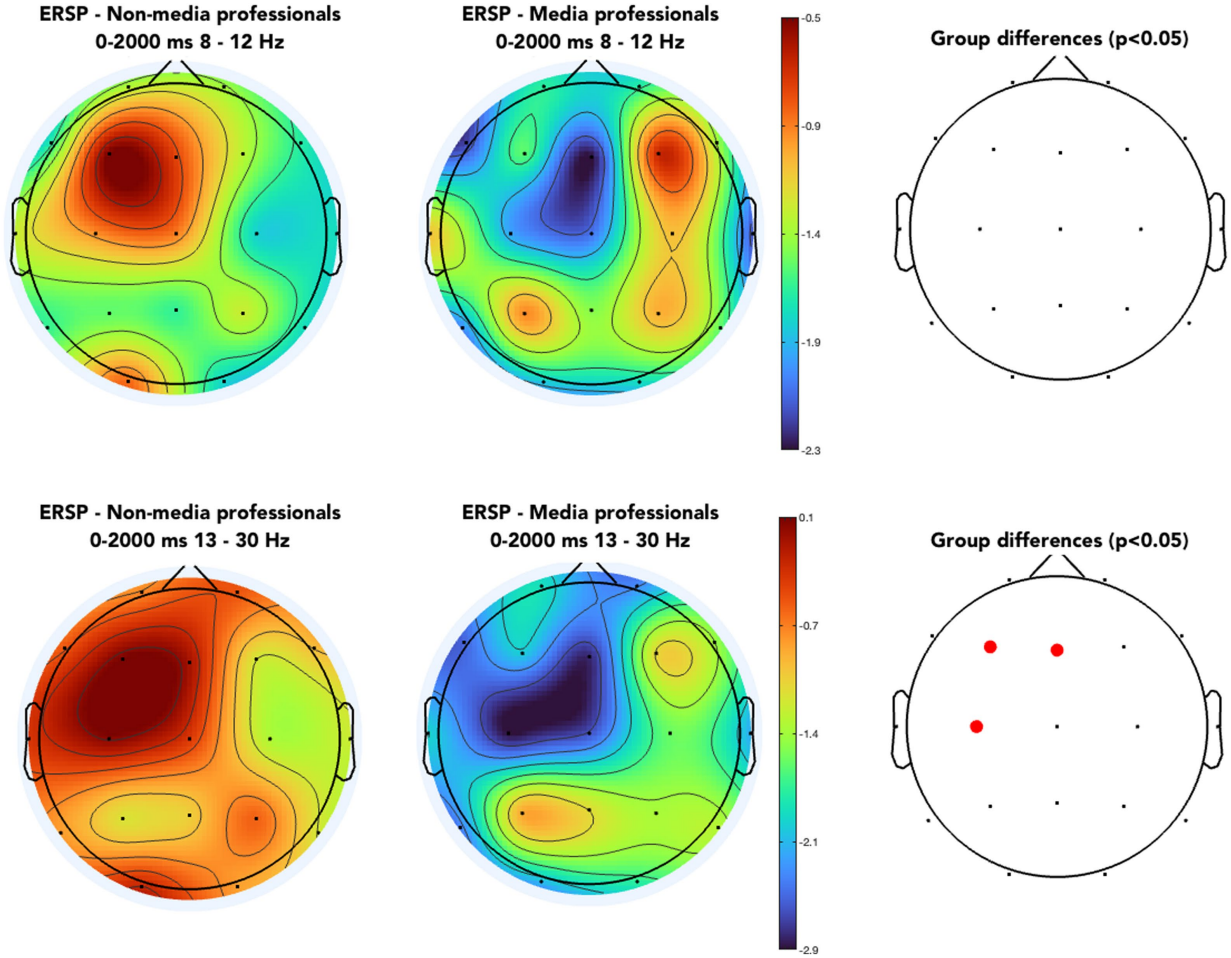
Average ERSPs of all electrodes in media and non-media professionals, from the onset of the motor activity to 2,000 ms after, in alpha (upper) and beta (lower). Red dots indicate significant differences found between groups (*p* < 0.05, *t*-test).

**Figure 4:**
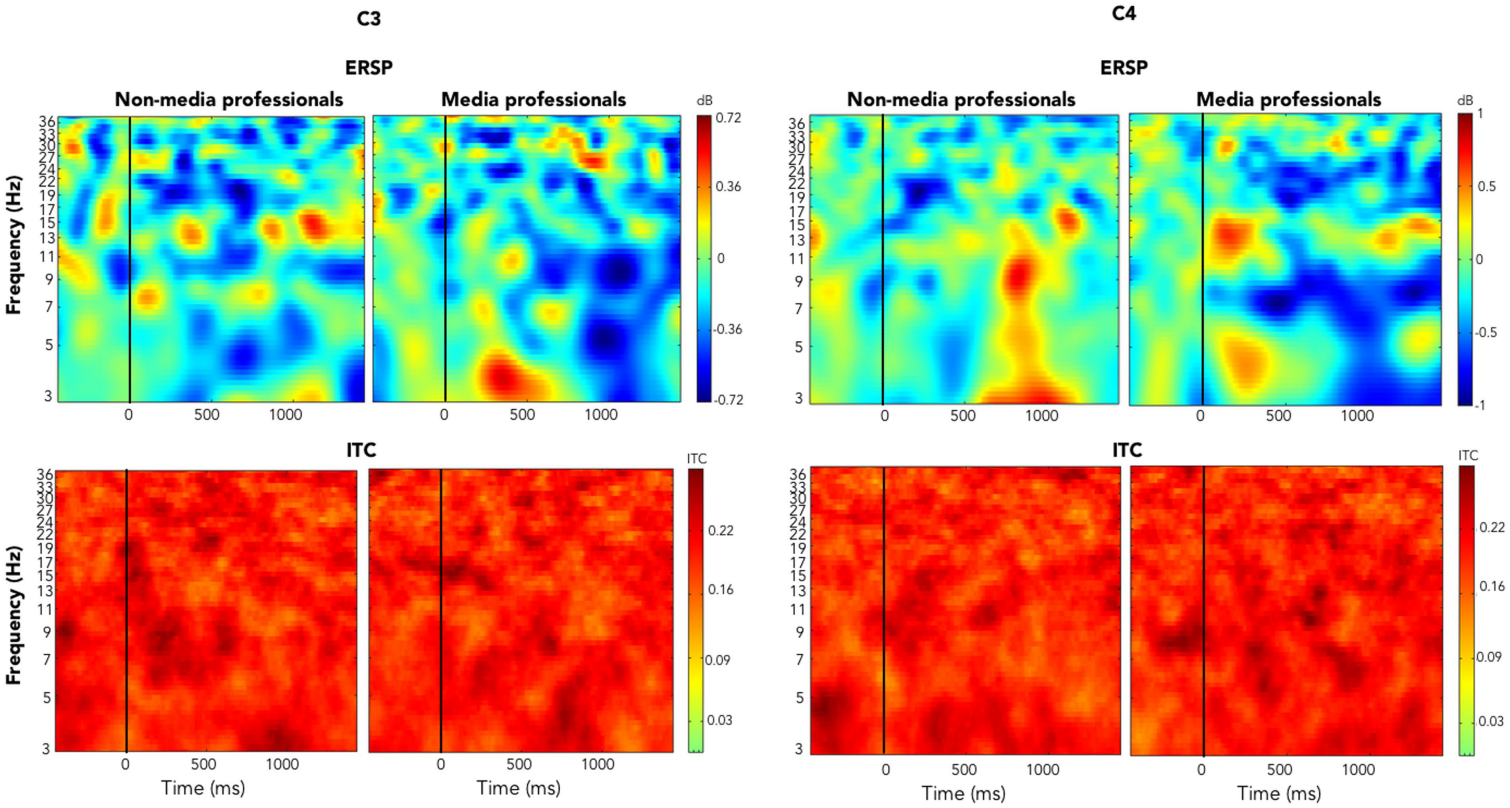
ERSPs (above) and ITC (below) in C3 and C4 in media and non-media professionals showing the temporal evolution. Vertical lines indicate the onset of the motor activity.

**Figure 5:**
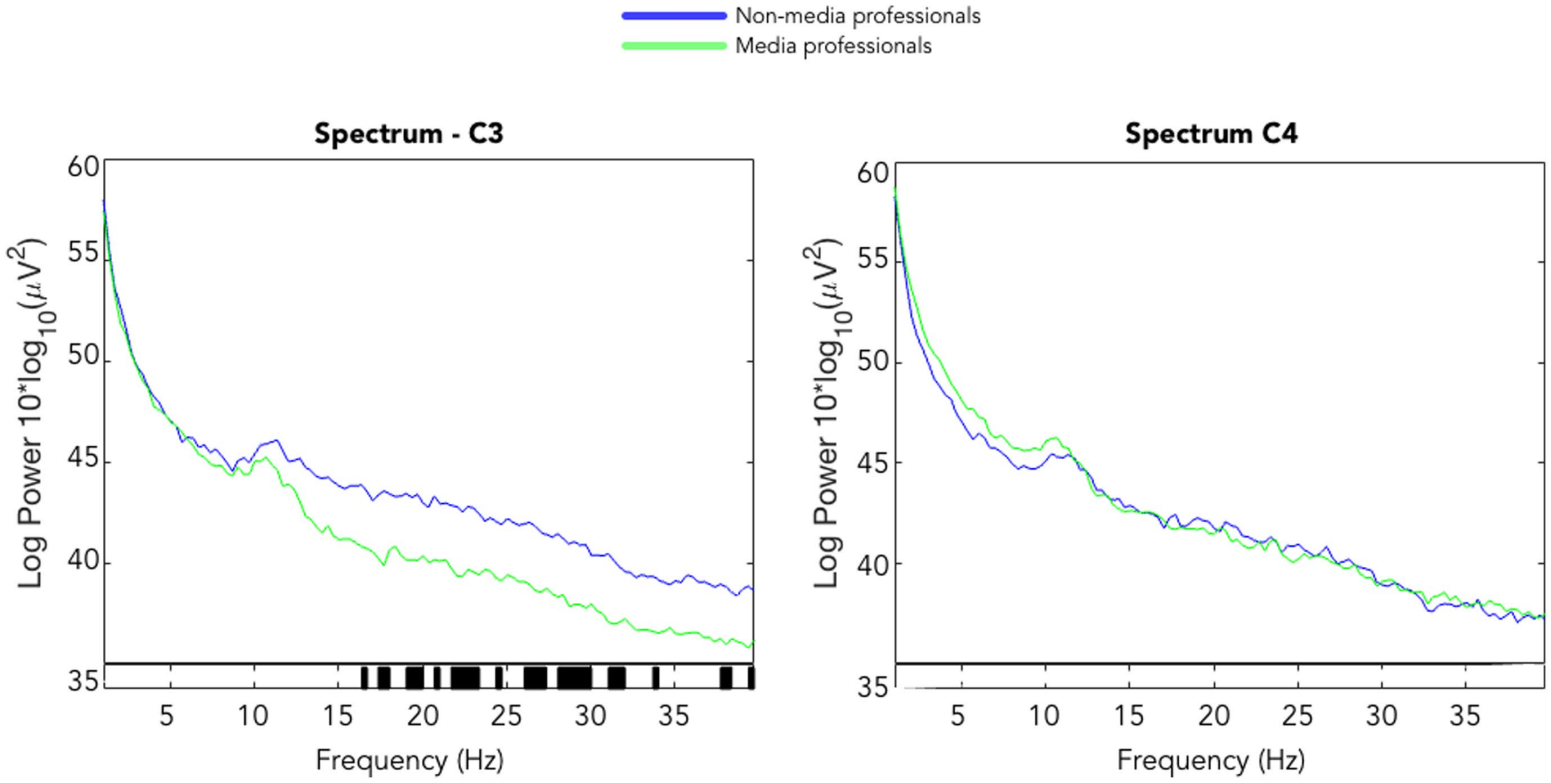
PSD of C3 and C4 activity in media (green) and non-media (blue) professionals in the whole 3-s epochs around the motor actions within the video stimulus. Significant differences (*p* < 0.05, *t*-test) between groups are shown in black on the *x*-axis.

When analyzing and comparing the ERSPs in C3 and C4 between the two groups, we found significant differences in the left hemisphere. Note that all trials included not a change of visual content but the onset of a motor action of the hand(s) of the character in scene (such as grasping an object), meaning that the visual presentation was in an organic flow, without any formal visual interruption, such as cut or flash. Media professionals decreased their activity around C3 significantly as compared with non-media professionals. Those differences were not found in the right hemisphere (see Figure 2). We also computed ERSPs in media and non-media professional groups, with an average of all electrodes, in alpha (8–12 Hz) and beta (13–30 Hz) bands, and compared them using a *t*-test to look for differences. No significant differences (*p* < 0.05) between groups were found in the alpha band. In the beta band, we found significant differences in motor cortex areas in the left hemisphere (around C3) but none in the right hemisphere (Figures 3, 4). When looking at differences in the PSD [Log Power 10*log_10_(μV^2^)] at C3 and C4, we also found statistically significant differences in the left hemisphere in the beta band—the group of non-media professionals showing a higher spectral power (Figure 5).

## 4. Discussion

Watching other people performing different motor actions modulates our brain processes. In part, films are based on the impact that artists’ and creators’ actions have on spectators. In recent years, neuroscientists have studied how looking at someone performing a motor action, such as grasping an object, modifies the brain activity of the observer (Babiloni et al., 2002). In fact, these changes in EEG activities have been linked to the mirror neuron system on several occasions (Muthukumaraswamy et al., 2004; Rizzolatti and Craighero, 2004; Perry and Bentin, 2009, 2010; Rizzolatti and Cattaneo, 2009; Marshall and Meltzoff, 2011; Ikeda et al., 2019). Moreover, some studies have proven that the imagination of a motor action also activates specific, complex brain networks (Cebolla et al., 2015, 2017). So far, these studies have been carried out in research centers around the world without, to our knowledge, paying any further attention to the media expertise of the perceiver. Here we proposed to analyze differences in brain activity when seeing someone doing motor activities in movies, based on whether the viewer had or did not have media professional expertise. Overall, we found significant differences in the left primary motor cortex (C3) in beta rhythm between the two groups, with a lower activity present in media professionals. In previous studies, beta oscillations have been correlated with planning and execution of grasping movements (Jasper and Penfield, 1949; Zaepffel et al., 2013; Khanna and Carmena, 2015), with a decrease in the spectral power of the oscillations during the preparation and the execution of voluntary movements. Beta-band desynchronization during motor preparation has been connected with the degree of uncertainty about the task (Tzagarakis et al., 2010). Here, we found that professional expertise of viewers when looking at someone doing motor actions impacts on that viewing with a decrease in the spectral power of the beta band as compared with the case of non-media professionals. It is difficult to understand the neural processes underlying these functional differences, but we think that perhaps media professionals are more impacted by the grasping actions since they might be more attentive to the narrative content, as we previously found (Andreu-Sánchez et al., 2017). It is true that the notable desynchronization in the motor area of the professionals (Figure 3) could be due to a more real sensation in this group in the narrative events of the movie. However, such desynchronization is also perceived prior to the onset of grasping actions. In fact, we have previously found that cuts in movies also provoke differences therein among both groups, showing a higher desynchronization in motor cortex in media professionals between 7 and 11 Hz at 200–300 ms after the cut (Andreu-Sánchez et al., 2021). In this regard, the reported results here would point to some important functional differences in the viewing of screens by media professionals. Although we initially found significant differences between media and non-media professionals in eye-blink rate when watching audiovisuals (Andreu-Sánchez et al., 2017), while studying differences between the two groups when they were looking at new visual information presented after audiovisual cuts, we found significant differences in frontal and parietal brain areas, but not in the occipital one (Andreu-Sánchez et al., 2021), suggesting that differences between these two groups might be more linked to the processing of the narrative content than to the actual visual processing of the formal visual information. Since everything points to a professionalizing effect in media professionals, it would be interesting to replicate previous studies regarding motor imagery paying attention to media professionalization as a variable (Cebolla et al., 2015) as it could have a big impact in brain-computer interface (BCI) training contexts. We also found sharp asymmetry (Figure 3) in alpha and beta bands, regardless the group. It coincides with previous works that suggest contralateral activity in human motor cortex correlated with the hand dominance, specialization, and activation (Hund-Georgiadis and von Cramon, 1999; Bai et al., 2005). Unfortunately, we did not ask participants regarding their hand dominance, which is a limitation of this work and something that could have improved the analysis of our results. Another limitation of our study is the unbalanced male–female sample which prevents us from analyzing sex as a solid variable here.

Our results could also be seen from a perception-action perspective. Perception-action approaches suggest that one of the most important aspects of motor control is predictive control (von Hofsten and Rosander, 2012) and it is based on experience (Ridderinkhof, 2014). Somehow, our brains use stored memories to constantly make predictions about what we see, feel, and hear (Hawkins and Blakeslee, 2004) and perception and actions would be unified through common principles (Ridderinkhof, 2014). In this context, the skill level has been previously correlated with perception processes linked with anticipatory tasks (Farrow and Abernethy, 2003). Here, we found that the professional experience (or expertise) in audiovisuals affects the brain activity in motor cortex while looking at motor actions. This suggests that the predictive control while viewing actions within the narrative contents could be trained by using audiovisuals as media professionals do on their daily basis.

## Data availability statement

The raw data supporting the conclusions of this article will be made available by the authors, without undue reservation.

## Ethics statement

The studies involving human participants were reviewed and approved by Ethics Commission for Research with Animals and Humans (CEEAH) of the Universitat Autònoma de Barcelona (Barcelona, Spain). The patients/participants provided their written informed consent to participate in this study.

## Author contributions

CA-S, MM-P, AG, and JD-G conducted the experimental design and wrote the article. CA-S and MM-P carried out experiments and data analyses. All authors contributed to the article and approved the submitted version.

## Funding

This study was supported by grant PID2021-122446NB-I00 funded by MCIN/AEI/10.13039/501100011033 and by “ERDF A way of making Europe” to AG and JD-G.

## Conflict of interest

The authors declare that the research was conducted in the absence of any commercial or financial relationships that could be construed as a potential conflict of interest.

## Publisher’s note

All claims expressed in this article are solely those of the authors and do not necessarily represent those of their affiliated organizations, or those of the publisher, the editors and the reviewers. Any product that may be evaluated in this article, or claim that may be made by its manufacturer, is not guaranteed or endorsed by the publisher.
